# SHARING ONE ANOTHER'S BURDENS: CONGREGATIONAL SUPPORT OF CAREGIVERS FOR PERSONS LIVING WITH DEMENTIA

**DOI:** 10.1093/geroni/igz038.1306

**Published:** 2019-11-08

**Authors:** Brianna Garrison

**Affiliations:** Baylor University, Houston, Texas, United States

## Abstract

This presentation will discuss the findings and implications from a mixed-methods study examining the impact of support services for caregivers of persons living with Dementia in their faith community. Caregivers and persons living with dementia participating in religious activities report numerous psychosocial benefits. Faith communities are the primary social network for older adults, with 48% of older adults attending religious services at least once weekly. Results will highlight specific opportunities for local congregations to foster spiritual connection and meaningful engagement with caregivers of persons living with Dementia. Findings will also describe key considerations and pathways for social work practitioners, researchers, and religious leaders to better serve older adults in their communities by providing education and strengths-based interventions in the context of local congregations. These findings have the potential to increase the reach of such programs to diverse, underserved populations.

